# Prognostic value of systemic immune-inflammation index in patients with gastric cancer

**DOI:** 10.1186/s40880-017-0243-2

**Published:** 2017-09-12

**Authors:** Kang Wang, Feiyu Diao, Zhijun Ye, Xinhua Zhang, Ertao Zhai, Hui Ren, Tong Li, Hui Wu, Yulong He, Shirong Cai, Jianhui Chen

**Affiliations:** 1 Gastrointestinal Surgery Center, The First Affiliated Hospital of Sun Yat-sen University, Guangzhou, 510080 Guangdong P. R. China; 2 Department of Gastrointestinal Surgery, Sun Yat-sen Memorial Hospital of Sun Yat-sen University, Guangzhou, 512120 Guangdong P. R. China; 3 Department of Gastroenterology and Hepatology, The First Affiliated Hospital of Sun Yat-sen University, Guangzhou, 510080 Guangdong P. R. China

**Keywords:** Gastric cancer, Preoperative systemic immune-inflammation index, Prognosis

## Abstract

**Background:**

Inflammation-based indexes have been used to predict survival and recurrence in cancer patients. Systemic immune-inflammation index (SII) was reported to be associated with prognosis in some malignant tumors. In the present study, we aimed to explore the association between SII and the prognosis of patients with gastric cancer.

**Methods:**

We retrospectively analyzed data from 444 gastric cancer patients who underwent gastrectomy at the First Affiliated Hospital of Sun Yat-sen University between January 1994 and December 2005. Preoperative SII was calculated. The Chi square test or Fisher’s exact test was used to determine the relationship between preoperative SII and clinicopathologic characteristics. Overall survival (OS) rates were estimated using the Kaplan–Meier method, and the effect of SII on OS was analyzed using the Cox proportional hazards model. Receiver operating characteristic (ROC) curves were used to compare the predictive ability of SII, NLR, and PLR.

**Results:**

SII equal to or higher than 660 was significantly associated with old age, large tumor size, unfavorable Borrmann classification, advanced tumor invasion, lymph node metastasis, distant metastasis, advanced TNM stage, and high carcino-embryonic antigen level, high neutrophil–lymphocyte ratio, and high platelet–lymphocyte ratio (all *P* < 0.05). High SII was significantly associated with unfavorable prognosis (*P* < 0.001) and SII was an independent predictor for OS (*P* = 0.015). Subgroups analysis further showed significant associations between high SII and short OS in stage I, II, III subgroups (all *P* < 0.05). SII was superior to NLR and PLR for predicting OS in patients with gastric cancer.

**Conclusion:**

Preoperative SII level is an independent prognostic factor for OS in patients with gastric cancer.

## Background

Gastric cancer is the fifth most common cancer and the second leading cause of cancer-related death worldwide [1]. It was estimated that 679,100 new cases of gastric cancer were diagnosed and 498,000 related deaths occurred in China in 2015 [2]. Despite the development of new surgical techniques and the use of chemotherapy and radiotherapy, gastric cancer is still an extremely deadly disease, and patients with gastric cancer have a generally unfavorable prognosis [3]. However, simple, low-cost, and effective methods in predicting the prognosis of patients with gastric cancer are still lacking, and it is important to identify a reliable biomarker to predict the prognosis.

Systemic inflammatory responses have been shown to involve in DNA damage, angiogenesis promotion, and tumor invasion and migration [4–7]. Moreover, a study indicated that circulating lymphocytes could reflect a patient’s inflammatory status [8]. Thus, some inflammation-based parameters, such as lymphocyte count, neutrophil–lymphocyte ratio (NLR), platelet–lymphocyte ratio (PLR), and systemic immune-inflammation index (SII), have been used to predict survival and recurrence in cancer patients [9–13]. SII, a parameter combining lymphocyte, neutrophil, and platelet counts, has shown to be more accurate than NLR and PLR in predicting the prognosis of patients with hepatocellular carcinoma [12] and small-cell lung cancer [13]. However, the significance of SII as a predictor of survival in patients with gastric cancer has not been assessed in our center. In this study, we aimed to evaluate the prognostic value of SII for survival in patients with gastric cancer who underwent surgery.

## Patients and methods

### Patients

We reviewed the records of patients with gastric cancer who underwent gastrectomy between January 1994 and December 2005 at the First Affiliated Hospital of Sun Yat-sen University in Guangzhou, China. All patients were diagnosed with pathologic examination. Patients were excluded if they met the following criteria: history of other malignant tumors, receipt of neoadjuvant therapy, lack of blood test results, lost to follow-up, lymphatic system disorders, acute coronary syndromes, valvular heart diseases, autoimmune thyroid diseases, and systematic inflammatory diseases. Tumor stage was defined according to the 2010 American Joint Committee on Cancer TNM staging system and the Japanese classification of gastric carcinoma, Borrmann classification.

### Clinical data collecting and processing

Baseline data, including demographic information, routine blood test results, tumor markers, and gastrectomy history, were reviewed. Following clinical and pathologic data were collected: sex, age, tumor location, tumor size, pathologic type, Borrmann classification, depth of invasion, lymph node metastasis, distant metastasis, and TNM stage.

SII, NLR, and PLR were calculated as follows: SII = P × N/L; NLR = N/L; and PLR = P/L, where P, N, and L stood for platelet, neutrophil, and lymphocyte counts, respectively [14, 15]. Optimal cutoff values of SII (low, < 660; high, ≥ 660); NLR (low, < 2.10; high, ≥ 2.10); and PLR (low, < 120; high, ≥ 120) were determined according to previous studies [14–16]. Cutoff values of age, tumor size, and carcino-embryonic antigen (CEA) were adopted from previous studies [17, 18].

### Follow-up

Patients were followed up every 3 months during the first year and every 6 months thereafter. Phone calls were made and letters were sent to patients and their relatives according to the National Comprehensive Cancer Network follow-up guidelines. Follow-ups were ended after December 2013.

### Statistical analysis

SPSS version 18.0 software (SPSS Inc., Chicago, IL, USA) was used for data analysis. Relationships between SII and clinicopathologic factors were analyzed using the Chi square test or Fisher’s exact test. OS was defined as the interval from gastrectomy to last follow-up or the date of death. Mean overall survival (OS) with 95% confidence interval (CI) were compared using the analysis of variance (ANOVA). Kaplan–Meier survival curves were constructed, and the log-rank test was used to compare survival rate. Censored data were used for patients who were alive at last follow-up or lost to follow-up. Univariate and multivariate analysis was performed using the Cox proportional hazards model. Variables in univariate analysis with *P* values less than 0.05 were included in multivariate analysis. Receiver operating characteristic (ROC) curves and area under the ROC curve (AUC) were used to assess the sensitivity and specificity of SII, NLR, and PLR in predicting the prognosis of patients with gastric cancer. For all analyses, *P* values less than 0.05 were considered statistically significant.

### Ethical approval

All procedures performed in studies involving human participants were in accordance with the ethical standards of institutional and/or national research committees and with the 1964 Helsinki Declaration, its later amendments, or similar ethical standards.

## Results

### Patient characteristics

The clinical and pathologic characteristics are shown in Table 1. In the present study, we included 444 patients; 281 (63.3%) were men and 163 (36.7%) were women. Median age was 56 years (range 21–87 years). Median follow-up duration was 45 months (range 1–185 months). At the last follow-up, 277 (62.4%) patients died, and 167 (37.6%) were still alive. Tumor location, tumor size, Borrmann classification, tumor invasion, lymph node metastasis, distant metastasis, TNM stage, CEA, and SII were significantly associated with OS of patients with gastric cancer (all *P* < 0.05).

**Table 1:** Clinicopathologic characteristics and overall survival information of 444 patients with gastric cancer who underwent gastrectomy

Characteristic	No. of patients (%)	Mean OS with 95% CI (months)	*P* value^b^
Sex			0.170
Man	281 (63.3)	54 (48–60)	
Woman	163 (36.7)	61 (53–69)	
Age (years)			0.061
<60	249 (56.1)	60 (53–66)	
≥60	195 (43.9)	53 (46–59)	
Tumor location			0.043
Upper stomach	140 (31.5)	48 (40–56)	
Middle stomach	137 (30.9)	58 (50–67)	
Lower stomach	167 (37.6)	62 (54–70)	
Tumor size (cm)			<0.001
<5	221 (49.8)	73 (66–80)	
≥5	223 (50.2)	40 (34–46)	
Pathologic type			0.330
Adenocarcinoma	367 (82.7)	57 (52–62)	
Squamous carcinoma	25 (5.6)	50 (28–71)	
Adenosquamous carcinoma	3 (0.7)	37 (18–57)	
Ring cell carcinoma	39 (8.8)	53 (36–69)	
Undifferentiated carcinoma	10 (2.3)	86 (39–133)	
Borrmann classification			<0.001
1	30 (6.8)	78 (55–102)	
2	98 (22.1)	76 (66–85)	
3	259 (58.3)	55 (49–61)	
4	50 (11.3)	20 (12–29)	
5	7 (1.6)	30 (3–55)	
pT^a^			<0.001
1	53 (11.9)	99 (87–110)	
2	41 (9.2)	89 (76–103)	
3	175 (39.4)	59 (52–65)	
4	175 (39.4)	34 (27–41)	
pN^a^			<0.001
0	152 (34.2)	92 (85–100)	
1	185 (41.7)	42 (36–48)	
2	77 (17.3)	34 (25–43)	
3	30 (6.8)	24 (12–37)	
pM^a^			<0.001
0	337 (75.9)	67 (63–73)	
1	107 (24.1)	21 (14–27)	
TNM stage^a^			<0.001
I	77 (17.3)	101 (92–110)	
II	64 (14.4)	79 (67–90)	
III	196 (44.1)	52 (45–58)	
IV	107 (24.1)	21 (14–27)	
CEA (μg/L)			0.023
<5	399 (89.9)	59 (54–63)	
≥5	45 (10.1)	39 (26–53)	
NLR			0.510
<2.10	205 (46.2)	65 (58–72)	
≥2.10	239 (53.8)	49 (43–56)	
PLR			0.201
<120	144 (32.4)	67 (59–76)	
≥120	300 (67.6)	51 (46–57)	
SII			<0.001
<660	283 (63.7)	65 (59–71)	
≥660	161 (36.3)	42 (35–49)	

OS, overall survival; CI, confidence interval; CEA, carcinoembryonic antigen; NLR, neutrophil–lymphocyte ratio; PLR, platelet–lymphocyte ratio; SII, systemic immune-inflammation index

^a^Tumor stage was defined according to the 2010 American Joint Committee on Cancer (AJCC) TNM staging system

^b^ANOVA analysis was used to compare the OS

### Relationship between SII and clinicopathologic characteristics

As shown in Table 2, high SII was associated with old age, large tumor size, unfavorable Borrmann classification, advanced tumor invasion, lymph node metastasis, distant metastasis, advanced TNM stage, high CEA level, high NLR, and high PLR (all *P* < 0.05).

**Table 2:** Association of SII with clinicopathologic characteristics in patients with gastric cancer

Characteristic	SII < 660 [cases (%)]	SII ≥ 660 [cases (%)]	*Χ* ^*2*^ value	*P* value
Total	283	161		
Sex			0.051	0.821
Men	178 (62.9)	103 (64.0)		
Women	105 (37.1)	58 (36.0)		
Age (years)			9.250	0.002
<60	174 (61.5)	75 (46.6)		
≥60	109 (38.5)	86 (53.4)		
Tumor location			5.667	0.059
Upper stomach	82 (29.0)	58 (36.0)		
Middle stomach	83 (29.3)	54 (33.5)		
Lower stomach	118 (41.7)	49 (30.4)		
Tumor size (cm)			20.867	<0.001
<5	164 (58.0)	57 (35.4)		
≥5	119 (42.0)	104 (64.6)		
Pathologic type			4.642	0.326
Adenocarcinoma	236 (83.4)	131 (81.4)		
Squamous carcinoma	12 (4.2)	13 (8.1)		
Adenosquamous carcinoma	3 (1.1)	0 (0.0)		
Ring cell carcinoma	26 (9.2)	13 (8.1)		
Undifferentiated carcinoma	6 (2.1)	4 (2.5)		
Borrmann classification			10.079	0.039
1	15 (5.3)	15 (9.3)		
2	74 (26.1)	24 (14.9)		
3	161 (56.9)	98 (60.9)		
4	28 (9.9)	22 (13.7)		
5	5 (1.8)	2 (1.2)		
pT			23.504	<0.001
1	46 (16.3)	7 (4.3)		
2	30 (10.6)	11 (6.8)		
3	115 (40.6)	60 (37.3)		
4	92 (32.5)	83 (51.6)		
pN			12.925	0.005
0	114 (40.3)	38 (23.6)		
1	109 (38.5)	76 (47.2)		
2	43 (15.2)	34 (21.1)		
3	17 (6.0)	13 (8.1)		
pM			36.571	<0.001
0	241 (85.2)	96 (59.6)		
1	42 (14.8)	65 (40.4)		
TNM stage			40.848	<0.001
I	62 (21.9)	15 (9.3)		
II	47 (16.6)	17 (10.6)		
III	132 (46.6)	64 (39.7)		
IV	42 (14.8)	65 (40.4)		
CEA (μg/L)			4.778	0.029
<5	261 (92.2)	138 (85.7)		
≥5	22 (7.8)	23 (14.3)		
NLR			52.974	<0.001
<2.10	197 (69.6)	8 (5.0)		
≥2.10	86 (30.4)	153 (95.0)		
PLR			86.897	<0.001
<120	137 (48.4)	7 (4.3)		
≥120	146 (51.6)	154 (95.7)		

OS, overall survival; CI, confidence interval; CEA, carcinoembryonic antigen; NLR, neutrophil–lymphocyte ratio; PLR, platelet–lymphocyte ratio; SII, systemic immune-inflammation index

### Association of SII with OS

**Fig. 1:**
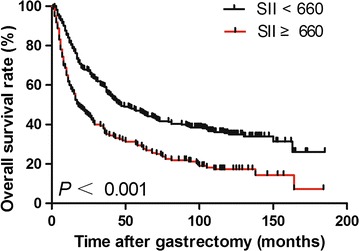
Kaplan–Meier overall survival (OS) curves for gastric cancer patients with high and low systemic immune-inflammation index (SII). Two curves were compared using log-rank test

Patients were divided into two groups according to SII values. Five-year OS rates were 46.1% and 29.4% in the group with SII lower than 660 and in the group with SII equal to or higher than 660, respectively. Median OS was significantly longer in patients with low SII than those with high SII (65 months, 95% CI 40–90 months vs. 22 months, 95% CI 14–30 months; *P* < 0.001; Fig. 1).

**Fig. 2:**
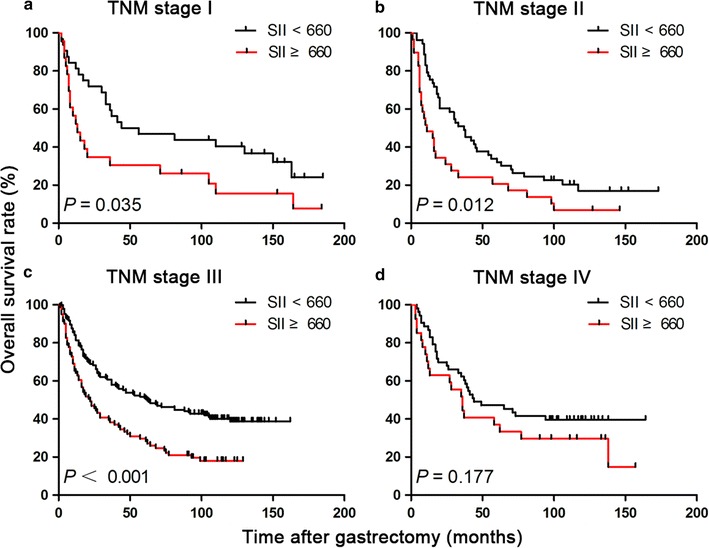
Kaplan-Meier OS curves for patients with high and low SII stratified by TNM stages. OS of patients with **a** TNM stage I; **b** TNM stage II; **c** TNM stage III; and **d** TNM stage IV gastric cancer

We further assessed the prognostic value of SII in different TNM stage groups. The results showed that preoperative SII was a prognostic indicator in patients with stage I (*P* = 0.035; Fig. 2a), stage II (*P* = 0.012; Fig. 2b), and stage III (*P* < 0.001; Fig. 2c) gastric cancer. However, for patients with stage IV gastric cancer, no significant association of SII with OS was identified (*P* = 0.177; Fig. 2d).

### Univariate and multivariate Cox regression analysis for OS

As shown in Table 3, in univariate analysis, age, tumor location, Borrmann classification, TNM stage, CEA, SII, NLR, and PLR were significant prognostic factors (all *P* < 0.05), whereas sex (*P* = 0.420) and pathologic type (*P* = 0.758) were not significant prognostic factors.

**Table 3:** Univariate and multivariate analyses of clinicopathologic factors and SII for OS of patients with gastric cancer

Variable	Univariate analysis		Multivariate analysis	
	HR (95% CI)	*P* value	HR (95% CI)	*P* value
Sex	0.904 (0.707–1.155)	0.420		NI
Age	1.301 (1.027–1.647)	0.029	1.356 (1.059–1.735)	0.026
Tumor location	0.766 (0.672–0.873)	<0.001	0.874 (0.762–1.003)	0.056
Tumor size	2.421 (1.897–3.092)	<0.001	1.290 (0.997–1.670)	0.017
Pathologic type	0.984 (0.886–1.092)	0.758		NI
Borrmann classification	1.790 (1.524–2.103)	<0.001	1.521 (1.279–1.810)	<0.001
TNM stage	2.428 (2.087–2.825)	<0.001	2.081 (1.772–2.443)	<0.001
CEA	1.844 (1.303–2.609)	0.001	1.431 (1.006–2.035)	0.027
NLR	1.581 (1.250–2.001)	<0.001	0.902 (0.637–1.278)	0.563
PLR	1.738 (1.332–2.267)	<0.001	1.272 (0.902–1.794)	0.170
SII	1.848 (1.455–2.348)	<0.001	1.551 (1.211–1.987)	0.015

HR, hazard ratio; NI, not included; OS, overall survival; CI, confidence interval; CEA, carcinoembryonic antigen; NLR, neutrophil–lymphocyte ratio; PLR, platelet–lymphocyte ratio; SII, systemic immune-inflammation index

Significant variables in univariate analysis were included in multivariate Cox regression analysis. We found that OS was independently associated with age (HR = 1.356, 95% CI 1.059–1.735, *P* = 0.026), Borrmann classification (HR = 1.521, 95% CI 1.279–1.810, *P* < 0.001), TNM stage (HR = 2.081, 95% CI 1.772–2.443, *P* < 0.001), CEA (HR = 1.431, 95% CI 1.006–2.035, *P* = 0.027), and SII (HR = 1.551, 95% CI 1.211–1.987, *P* = 0.015), whereas tumor location, NLR, and PLR were not independent prognostic factors.

### Comparison between inflammation indexes

**Fig. 3:**
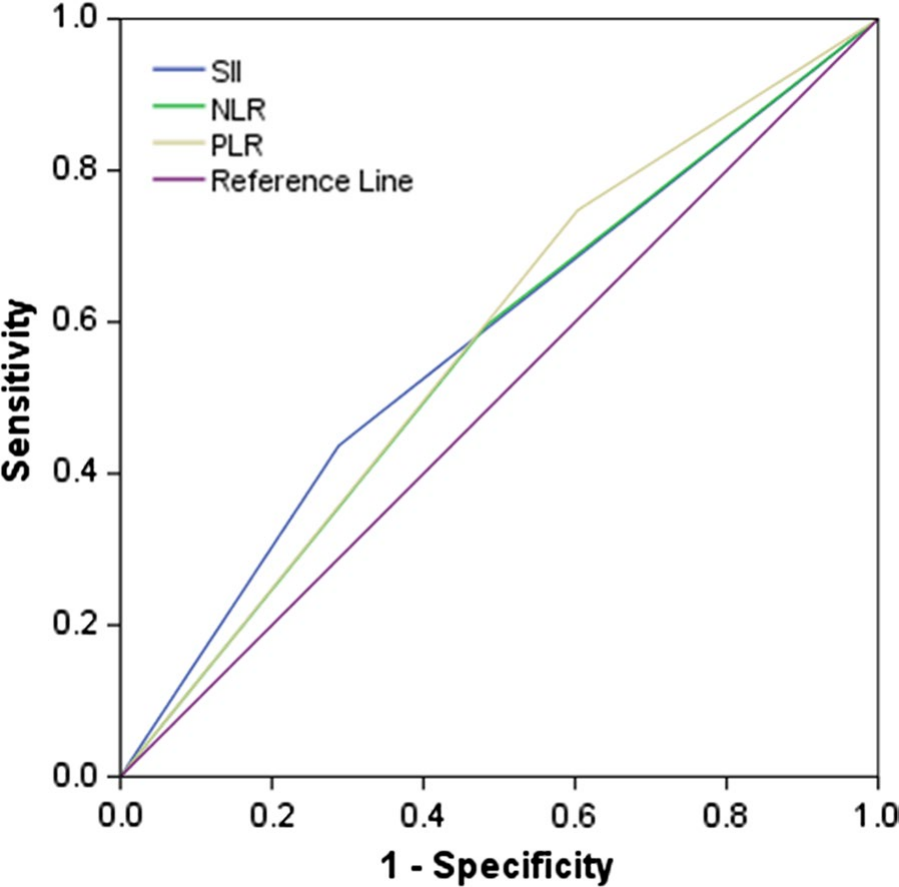
Predictive ability of SII compared with NLR and PLR by receiver operating characteristic (ROC) curve analysis. NLR, neutrophil–lymphocyte ratio; PLR, platelet–lymphocyte ratio

We evaluated the prognostic values of these systemic inflammation indexes. Area under the ROC curve (AUC) of SII, NLR, and PLR were 0.612 (*P* = 0.003), 0.556 (*P* = 0.040), and 0.572 (*P* = 0.009), respectively (Fig. 3). These results indicated that SII was superior to NLR and PLR for predicting OS in patients with gastric cancer.

## Discussion

In the present study, we found that high SII was an independent predictor of poor prognosis in patients with stage I–III gastric cancer, and SII was a superior prognostic index compared with NLR and PLR.

We found that, in patients with gastric cancer, high SII was associated with old age, large tumor size, poor Borrmann classification, advanced tumor invasion, lymph node metastasis, distant metastasis, advanced TNM stage, and high CEA level. It has recently been shown that systemic inflammatory responses induce malignant tumor behavior and are associated with short survival in patients with various malignant solid tumors and that systemic inflammation indexes can predict cancer prognosis [19–23]. Hu et al. [12] showed that high SII was associated with liver cirrhosis, low tumor differentiation, large tumor size, early recurrence, and high circulating tumor cell levels in patients with hepatocellular carcinoma. Moreover, Hong et al. [13] found that high SII was associated with sex and hemoglobin level in patients with small cell lung cancer.

Preoperative inflammation indexes were associated with OS in many cancers. Peng et al. [23] found that high PLR was strongly associated with poor outcome in patients with metastatic colorectal cancer. Ji et al. [11] found that preoperative NLR and PLR were significant predictors of OS in patients with esophageal squamous cell carcinoma and that PLR was superior to NLR as a prognostic index. We also found SII was an independent prognostic index in patients with gastric cancer. In addition, we found that SII was a better predictor than NLR and PLR.

TNM stage is the gold standard for predicting the OS of patients with gastric cancer. However, TNM stage is determined postoperatively. Therefore, preoperative prognostic prediction can be difficult. SII is a convenient, easily obtained, low-cost, and non-invasive biomarker that is a complement to TNM stage as a prognostic predictor for patients with gastric cancer. Moreover, we found that SII was a strong prognostic index for patients with stage I–III gastric cancer. As for patients with stage IV gastric cancer, OS was shorter in patients with high SII level. However, the difference was not significant, and maybe the cutoff value needs to be optimized.

Our study had several limitations. First, it was a single-center, retrospective study with a relatively small sample size. Thus, conclusions from the present study may have a bias. Second, although SII was an independent predictor of gastric cancer prognosis and was superior to NLR and PLR, the sensitivity and specificity of SII was not very high, indicating that perspective studies to find a proper cutoff value are needed.

## Conclusions

We found that preoperative SII was a simple, strong, and independent predictor of OS in patients with gastric cancer. However, additional studies are needed to verify its prognostic value.

## References

[1] Lindsey A, Torre M, Freddie Bray P, Siegel RL, Jacques Ferlay M (2015). Global cancer statistics, 2012. CA Cancer J Clin.

[2] Chen W, Zheng R, Baade PD, Zhang S, Zeng H, Bray F (2016). Cancer Statistics in China, 2015. CA Cancer J Clin.

[3] Ma J, Yao S, Li X, Kang H, Yao F, Du N (2015). Neoadjuvant therapy of DOF regimen plus bevacizumab can increase surgical resection rate in locally advanced gastric cancer. Medicine.

[4] Hernández M, Martín R, Garcíacubillas MD, Maesohernández P, Nieto ML (2010). Secreted PLA2 induces proliferation in astrocytoma through the EGF receptor: another inflammation-cancer link. Neuro-Oncology..

[5] Nguyen AV, Wu YY, Liu Q, Wang D, Nguyen S, Loh R (2013). STAT3 in epithelial cells regulates inflammation and tumor progression to malignant state in colon. Neoplasia..

[6] Nguyen AV, Wu YY, Lin EY (2014). STAT3 and sphingosine-1-phosphate in inflammation-associated colorectal cancer. World J Gastroenterol.

[7] Dai J, Lu Y, Roca H, Keller JM, Zhang J, McCauley LK (2017). Immune mediators in the tumor microenvironment of prostate cancer. Chin J Cancer..

[8] Wang L, Shen Y (2013). Imbalance of circulating T-lymphocyte subpopulation in gastric cancer patients correlated with performance status. Clin Lab..

[9] Li S, Xu X, Liang D, Tian G, Song S, He Y (2014). Prognostic value of blood neutrophil-to-lymphocyte ratio (NLR) and platelet-to-lymphocyte ratio (PLR) in patients with gastric cancer. Zhonghua Zhong Liu Za Zhi..

[10] Lian L, Xia YY, Zhou C, Shen XM, Li XL, Han SG (2015). Application of platelet/lymphocyte and neutrophil/lymphocyte ratios in early diagnosis and prognostic prediction in patients with resectable gastric cancer. Cancer Biomark..

[11] Feng JF, Huang Y, Chen QX (2014). Preoperative platelet lymphocyte ratio (PLR) is superior to neutrophil lymphocyte ratio (NLR) as a predictive factor in patients with esophageal squamous cell carcinoma. World J Surg Oncol..

[12] Hu B, Yang XR, Xu Y, Sun YF, Sun C, Guo W (2014). Systemic Immune-Inflammation index predicts prognosis of patients after curative resection for hepatocellular carcinoma. Clin Cancer Res.

[13] Hong X, Cui B, Wang M, Yang Z, Wang L, Xu Q (2015). Systemic immune-inflammation index, based on platelet counts and Neutrophil-Lymphocyte ratio, is useful for predicting prognosis in small cell lung cancer. The Tohoku J Exp Med.

[14] Yang Z, Zhang J, Lu Y, Xu Q, Tang B, Wang Q (2015). Aspartate aminotransferase-lymphocyte ratio index and systemic immune-inflammation index predict overall survival in HBV-related hepatocellular carcinoma patients after transcatheter arterial chemoembolization. Oncotarget..

[15] Aldemir MN, Turkeli M, Simsek M, Yildirim N, Bilen Y, Yetimoglu H (2015). Prognostic value of baseline neutrophil-lymphocyte and platelet-lymphocyte ratios in local and advanced gastric cancer patients. Asian Pac J Cancer Prev.

[16] Sun X, Liu X, Liu J, Chen S, Xu D, Li W (2016). Preoperative neutrophil-to-lymphocyte ratio plus platelet-to-lymphocyte ratio in predicting survival for patients with stage I-II gastric cancer. Chin J Cancer..

[17] Sun Z, Zhang N (2014). Clinical evaluation of CEA, CA19-9, CA72-4 and CA125 in gastric cancer patients with neoadjuvant chemotherapy. World J Surg Oncol..

[18] Lu J, Huang CM, Zheng CH, Li P, Xie JW, Wang JB (2013). Consideration of tumor size improves the accuracy of TNM predictions in patients with gastric cancer after curative gastrectomy. Surg Oncol.

[19] Lin C, Lin W, Yeh S, Li L, Chang C (2015). Infiltrating neutrophils increase bladder cancer cell invasion via modulation of androgen receptor (AR)/MMP13 signals[J]. Oncotarget.

[20] Galvao RP, Zong H (2013). Inflammation and gliomagenesis: Bi-Directional communication at early and late stages of tumor progression. Curr Pathobiol Rep..

[21] Wong CE, Yu JS, Quigley DA, To MD, Jen KY, Huang PY (2013). Inflammation and Hras signaling control epithelial-mesenchymal transition during skin tumor progression. Genes Dev.

[22] Tsai PL, Su WJ, Leung WH, Lai CT, Liu CK (2016). Neutrophil-lymphocyte ratio and CEA level as prognostic and predictive factors in colorectal cancer: a systematic review and meta-analysis. J Cancer Res Ther..

[23] Peng HX, Lin K, He BS, Pan YQ, Ying HQ, Hu XX (2016). Platelet-to-lymphocyte ratio could be a promising prognostic biomarker for survival of colorectal cancer: a systematic review and meta-analysis. FEBS Open Bio.

